# Lung Cancer Pre-Diagnostic Pathways from First Presentation to Specialist Referral

**DOI:** 10.3390/curroncol28010040

**Published:** 2021-01-11

**Authors:** Satya Rashi Khare, Sreenath Arekunnath Madathil, Gerald Batist, Peter Brojde Lung Cancer Group, Isabelle Vedel

**Affiliations:** 1 Department of Family Medicine, Faculty of Medicine, McGill University, Montreal, QC H3S 1Z1, Canada; isabelle.vedel@mcgill.ca; 2 Division of Oral and Maxillofacial Surgery, Faculty of Dentistry, McGill University, Montreal, QC H3A 1G1, Canada; sreenath.madathil@mcgill.ca; 3 Department of Oncology, Segal Cancer Centre, Jewish General Hospital, Montreal, QC H3T 1E2, Canada; gerald.batist@mcgill.ca; 4 Peter Brojde Lung Cancer Centre, Jewish General Hospital, Montreal, QC H3T 1E2, Canada; 5 Lady Davis Institute, Jewish General Hospital, Montreal, QC H3T 1E2, Canada

**Keywords:** primary care, lung cancer, diagnostic pathways, early diagnosis, delay

## Abstract

Background: Lung cancer is often diagnosed at a late stage with high associated mortality. Timely diagnosis depends on timely referral to a respiratory specialist; however, in Canada, little is known about how patients move through primary care to get to a respiratory specialist. Accordingly, we aimed to identify and describe lung cancer pre-diagnostic pathways in primary care from first presentation to referral. Methods: In this retrospective cohort study, patients with primary lung cancer were recruited using consecutive sampling (*n* = 50) from a lung cancer center in Montréal, Québec. Data on healthcare service utilization in primary care were collected from chart reviews and structured patient interviews and analyzed using latent class analysis to identify groups of patients with similar pre-diagnostic pathways. Each group was described based on patient- and tumor-related characteristics and the sequence of utilization activities. Results: 68% of the patients followed a pathway where family physician (FP) visits were dominant (“FP-centric”) and 32% followed a pathway where walk-in clinic and emergency department (ED) visits were dominant (“ED-centric”). Time to referral in the FP group was double that of the ED group (45 days (IQR: 12–111) vs. 22 (IQR: 5–69)) with more advanced disease (65% vs. 50%). In the FP group, 29% of the patients saw their FP three times or more before being referred and 41% had an ED visit. Conclusions: Our findings may reflect the challenge of diagnosing lung cancer in primary care, missed opportunities for earlier diagnosis, and a lack of integration between primary and specialist care.

## 1. Introduction

In Canada, lung cancer represents 13% of new cancer cases and 26% of cancer deaths making it the most commonly diagnosed cancer and the leading cause of cancer-related mortality [1]. With a five-year survival of 17%, lung cancer kills more people than all other common cancers combined [2]. The most important prognostic factor is stage at diagnosis with treatment being more successful in early-stage disease. However, 70% of Canadians with lung cancer are diagnosed with late-stage disease [3], emphasizing the need to improve timely diagnosis.

Most lung cancer patients initially present to their family physician with symptoms [4,5], making the primary care interval a key component of the diagnostic interval [6]. This interval spans from first presentation with signs and symptoms suggestive of lung cancer to referral to a respiratory specialist. Despite this, Canadian research on reducing time to diagnosis has been concentrated in secondary care—from referral to definitive diagnosis—leading to an evidence gap on delays in timely referral [7]. This is concerning as studies in countries with similar healthcare systems (i.e., gatekeeper systems) have shown longer delays in primary care [5], some as much as four times greater than those observed in secondary care [8]. A major reason is that common presenting symptoms in primary care like cough have low positive predictive values for lung cancer, while symptoms with high positive predictive values like hemoptysis are rare [9,10].

Importantly, rapid diagnosis can be associated with worse survival. Known as the waiting time paradox, late-stage disease may present serious symptoms that lead to quicker investigation and shortened diagnostic times, but also poor outcomes [11]. This complicated association between timely diagnosis and survival suggests the time to diagnosis may not be as important as the path to diagnosis which should be without unnecessary delays. In fact, variation in how patients are managed in primary care has been suggested to contribute to international cancer survival differences [12].

Among Canadian provinces, Québec has the highest lung cancer incidence and mortality rates [13], yet there are no studies on lung cancer pre-diagnostic pathways in primary care and, as a result, no provincewide primary care initiatives aimed at reducing unnecessary delays. The lack of knowledge in Canada, especially Québec, on how lung cancer patients move through primary care has made it difficult to inform practice improvements. In order to ground improvement initiatives in local contexts, extensive research in primary care is needed.

In this study, we aimed to gain an in-depth understanding of lung cancer pre-diagnostic pathways in primary care in Québec in order to inform potential improvement initiatives. Firstly, we identified different pathways by clustering patients with similar patterns of healthcare utilization in the primary care interval into distinct groups. Secondly, we examined patient and clinical characteristics of each group as well as the sequence of healthcare utilization activities. This detailed understanding was then used to suggest several improvement strategies.

## 2. Methods

### 2.1. Study Design and Population

This retrospective cohort study took place at the Peter Brojde Lung Cancer Center (PBLCC) located in a large teaching hospital in Montréal. The clinic serves approximately 200 new lung cancer patients annually and maintains a detailed patient registry. We included patients diagnosed with primary lung cancer between 1 May 2015 and 31 October 2017. We excluded patients if they were actively followed in pulmonology for another respiratory condition at the time of referral or if their cancer was discovered incidentally; in both scenarios, the pre-diagnostic pathway generally does not involve primary care. We also excluded patients who were presenting for a second opinion due to incomplete data within the study setting.

We recruited patients by consecutive sampling. A list of eligible patients was pulled from the PBLCC registry and contacted by trained research assistants and nurses during a clinic appointment or by phone to ensure the most exhaustive sample. Two research assistants were present at every clinic (five per week) to approach patients in person and two nurses made several attempts to contact patients by phone. For patients who agreed to participate, we further extended the invitation to a family member who was knowledgeable about use of healthcare services during the primary care interval.

### 2.2. Data Collection

In accordance with methodological recommendations for early cancer diagnosis research [6], we used three data sources: the PBLCC registry was used to collect demographic and tumor-related data, patient charts were used to collect documented healthcare service utilization data during the primary care interval, and patient interviews were used to complete the account of pre-diagnostic activities.

We collected the following data from the registry: age at diagnosis, sex, referral source, referral date, diagnosis date, and stage of disease. Stages I and II were categorized as early, stage III as locoregional, and stage IV as advanced [14]. Additionally, postal codes were used to convert to an area-based deprivation score for each patient [15].

We collected the following utilization data from chart reviews and structured patient interviews: number of family physician visits, walk-in clinic visits, emergency department visits, hospitalizations, chest radiographs, computed tomography scans, and non-respiratory specialist visits. Additionally, presenting symptoms, comorbidities, and smoking history were abstracted from charts. The date of first presentation in primary care, set at a maximum of one year prior to the date of referral, was collected at the interview. The purpose of using multiple data sources was twofold. First, secondary care charts do not have complete data on primary care visits nor are they detailed enough to discern complex timepoints such as the date of first presentation [6]. This necessitated patient interviews. Second, validation studies of self-reported healthcare utilization emphasize underreporting, especially in the context of primary care visits [16]. By cross-verifying chart and interview data, underreporting was mitigated and data were more complete.

**Figure 1:**
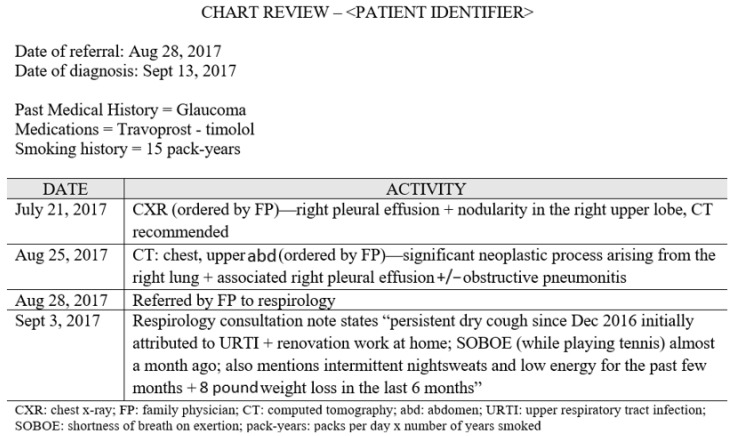
Chart review conducted on a patient from one year prior to their referral date to one month after their diagnosis date.

Patient charts were comprehensively reviewed from one year prior to the referral date to one month post-diagnosis by a researcher with extensive chart review experience in the study setting (S.K.). Information pertaining to the primary care interval was documented as a timeline in ascending order (Figure 1). All data from the chart review were verified during the interviews.

**Figure 2:**
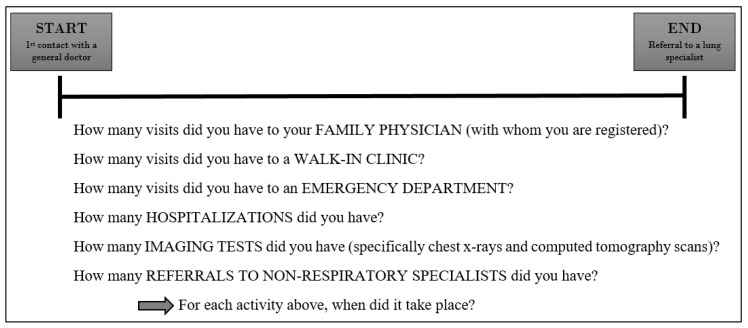
Cue card given to patients and caregivers in advance of the interview.

**Figure 3:**
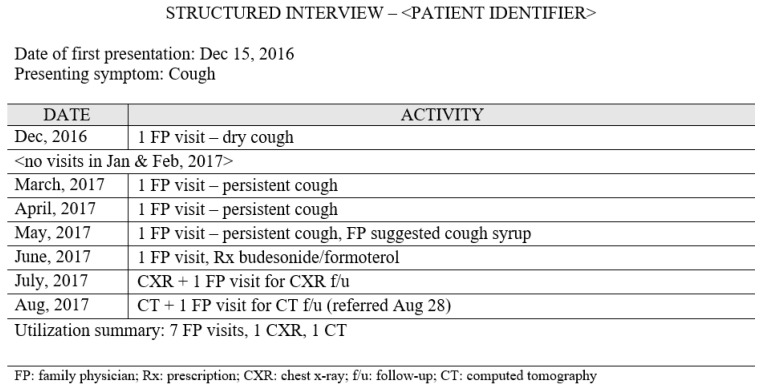
Summary of the interview data shown as the number of healthcare utilization activities by month.

Patient interviews were conducted in a private room at the clinic or the patient’s home by two trained research assistants who pilot tested the interview guide (Supplementary Materials) to ensure clarity and precision of questions. Before the interview, we gave patients a cue card (Figure 2) with information on the data that would be collected. During the interview, imaging data (date- and time-stamped documents) from the chart review were used to ground patients in the time period of interest. Several measures were used to improve event recollection including memory aids, forward recall, and use of large calendar sheets for collaborative data recording. These measures are described in detail elsewhere [17]. As an example of the additional data provided, Figure 3 shows a summary of interview data for the patient whose chart review is shown in Figure 1.

### 2.3. Statistical Analysis

We analyzed healthcare utilization data using latent class analysis (LCA), a model-based approach to clustering that statistically partitions a heterogeneous population (lung cancer patients) into homogeneous subgroups (pre-diagnostic pathways) according to response patterns to observed variables (utilization activities) [18]. The subgroups are latent classes that represent unobservable categorical constructs inferred indirectly through observed variables. Therefore, this analysis is most useful when the construct of interest is unobservable, unmeasurable, and unknown, as is the case with pre-diagnostic pathway groups [19].

As the distribution of utilization variables ranged from 0–2, we dichotomized them as none (0) versus any (1+). In the modeling process, we started with a one-class solution and added classes in a progressive fashion up to four classes. We evaluated the resulting models statistically (model fit) using the Akaike information criterion (AIC) and substantively (model usefulness) according to the knowledge of the construct being modelled [20]. This was completed by family medicine researchers, cancer researchers, an oncologist, and a cancer epidemiologist over several workshops. We then described each class in the final model based on the distribution of demographic, patient-, and tumor-related characteristics. Finally, we performed an event sequence analysis where utilization activities were presented as events occurring at a given position thus showing their order [21]. All analyses were conducted using R version 3.5.

### 2.4. Ethics Approval

The Research Review Office of the Integrated Health and Social Services University Network for West–Central Montréal granted ethics approval (code CODIM-FLP-17-031).

## 3. Results

**Figure 4:**
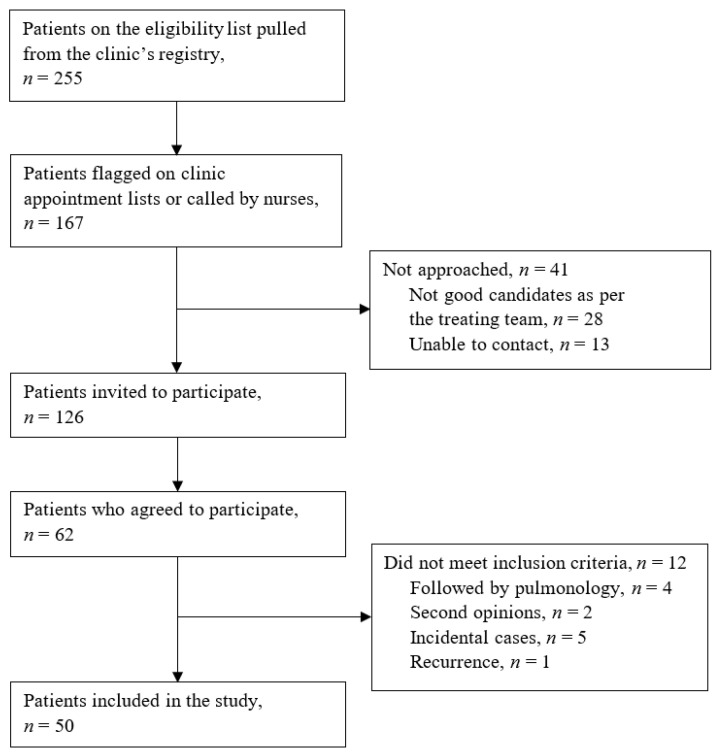
Flow of participant recruitment from the Peter Brojde Lung Cancer Center.

We recruited 62 patients between June and December 2017; 12 were later found to be ineligible leaving a total of 50 patients included in the study (Figure 4).

Patient characteristics are reported in Table 1. Most cases (90%) were non-small cell lung cancer, 36% of the patients had no comorbid conditions, 28% were never smokers, and 60% were advanced stage. Characteristics of the study sample were similar to registry patient characteristics except for smoking status where the study sample had almost double the number of never smokers [14].

The total diagnostic interval was a median of 82 days (IQR: 37–180). The primary care interval was a median of 35 days (IQR: 9–101) and the secondary care interval was a median of 27 days (IQR: 11–65).

### 3.1. Identifying Pre-Diagnostic Pathways

We identified a two-class model as the best fit model according to statistical and substantive criteria [20]. AIC pointed to a two- or three-class model as optimal (Table 2). Upon interpretation for meaning, the three-class model contained both classes found in the two-class model with a small third class (*n* = 6) that was not found to be meaningful after expert review.

**Figure 5:**
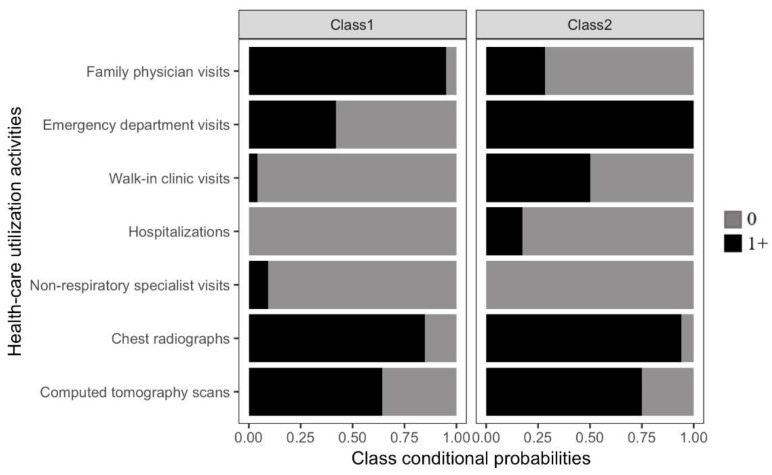
Class-conditional probabilities for the final two-class model from the latent class analysis.

The final class-conditional probabilities are presented in Figure 5. With a 68% (*n* = 34) prevalence, class 1 had 0.95 probability of family physician visits, 0.42 probability of emergency department visits, 0.04 probability of walk-in clinic visits, and zero probability of hospitalization. Given the high probability of at least one family physician visit, we labelled this class “Family physician (FP)-centric” pre-diagnostic pathway group.

With a 32% (*n* = 16) prevalence, class 2 had 0.28 probability of family physician visits, 1.0 probability of emergency department visits, 0.5 probability of walk-in clinic visits, and 0.17 probability of hospitalization. Given the high probability of at least one emergency department visit, we labelled this class “Emergency department (ED)-centric” pre-diagnostic pathway group.

Probability of imaging and non-respiratory specialist visits did not meaningfully differentiate the classes.

### 3.2. Characteristics of Patients by Pre-Diagnostic Pathway Group

Patient characteristics by group are reported in Table 3.

The FP-centric group had a primary care interval time of 45 days (IQR: 12–111) and 65% of the patients had advanced stage disease. In this group, 50% of the patients were referred to a respiratory specialist by their FP and 38% were referred from the ED. The most common presenting symptoms were cough and shortness of breath.

The ED-centric group had a primary care interval time of 22 days (IQR: 5–69) and 50% of the patients had advanced stage disease. In this group, 6% of the patients were referred to a respiratory specialist by their FP and 81% were referred from the ED. The most common presenting symptoms were cough and those categorized as “other”, including hoarseness and weight loss.

Other demographic and patient-related characteristics were similar between the groups.

### 3.3. Sequence of Events within Pre-Diagnostic Pathways

**Figure 6:**
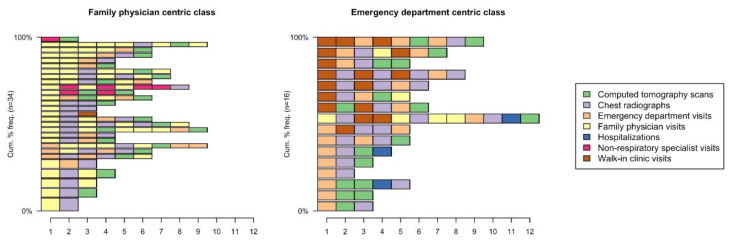
Sequence of utilization activities for each patient showing the order of specific events within the pathway stratified by group.

The sequence of utilization activities within each patient’s pathway is shown in Figure 6 by group. In the FP-centric group, 88% of the patients started their pathway with a visit to their FP. 62% of the patients had imaging after 1–2 FP visits. Throughout their entire pathway, 29% of the patients saw their FP three times or more before being referred and 41% of the patients had an ED visit. In this group, 68% of the pathways had a sequence of events that differed from all the other pathways (i.e., they were unique).

In the ED-centric group, 50% of the patients started their pathway with a visit to the ED and 44% of the patients started their pathway with a visit to a walk-in clinic. All the patients had imaging after 1–2 visits to the ED or walk-in clinic and all the patients had at least one ED visit in their pathway. In this group, none of the pathways shared the same sequence of events.

## 4. Discussion

This study presents findings on how lung cancer patients move through primary care in a province with high lung cancer incidence and mortality rates in order to inform early diagnosis initiatives. Median time in the primary care interval was not much greater than the secondary care interval (35 vs. 27 days); however, there was a large variation (9–101 days). Over 2/3 of the patients followed a pathway where FP visits were dominant (FP-centric) and less than 1/3 followed a pathway where walk-in clinic and ED visits were dominant (ED-centric). The FP-centric group had a primary care interval time that was double that of the ED-centric group (45 days (IQR: 12–111) vs. 22 (IQR: 5–69)) and more advanced stage disease (65% vs. 50%).

A large UK study similarly showed that patients who saw their FPs prior to diagnosis had significantly longer diagnostic intervals than those who did not [22]. In our study, we found that while 62% of the patients in the FP-centric group had imaging after 1–2 FP visits, 29% visited their FP three times or more before being referred to a respiratory specialist. Given that patients in this group mostly presented with cough and shortness of breath, this may be a reflection of the diagnostic difficulty associated with lung cancer [23] owing in part to non-specific symptoms [24]. Additionally, chest radiography is the principal diagnostic test in primary care, but has been shown to have a high false negative rate for lung cancer [25]. Alternatively, this could represent missed opportunities for earlier diagnosis with disease progression over time leading to the higher proportion of advanced stage disease found in this group [26]. In either case, more education in primary care on common presentations of lung cancer patients, including the risk threshold for referral, may contribute to reduced delays. One method could consist of significant event audits where performance feedback is used to prompt FPs to review their diagnostic practice and identify improvement opportunities [27]. Lessons learned could also be shared between primary care practices.

Despite the FP-centric group being dominated by FP visits, there was still moderate use of the ED. In this group, 38% of the patients were referred to a respiratory specialist from the ED with 41% of the patients visiting the ED at least once in their pathway. This could be linked to higher symptom severity or FPs may be using the ED as a means for quicker access to a specialist due to a lack of integration between primary and specialist care [28]. Rapid investigation clinics were implemented in Québec to fast-track diagnosis of patients with suspected lung cancer; however, our study suggests that these are not used as intended [29]. Québec also recently implemented an electronic referral system to improve access to specialists. While an impact evaluation has not yet been done, electronic consultation services where FPs can discuss cases with respiratory specialists before referral could further promote integration and reduce delays [30].

The ED-centric group in our study likely represents patients without an FP who use walk-in clinics and EDs for their primary care needs. Nationally, Québec has the highest number of persons without an FP [31]. Walk-in clinics were intended to reduce ED burden; however, our study found that all the patients in this group had an ED visit. This suggests that walk-in clinics may be ineffective at reducing ED visits and supports the need for improved access to a regular source of care. More nurse practitioners in the primary care setting could help fill the gap as Québec currently employs the lowest number in Canada [32].

Importantly, increasing the number of primary care practitioners alone will likely not resolve access issues. We found a considerably shorter primary care interval time in the ED-centric group that suggests greater care efficiency among patients who present at the ED. In accordance with the waiting time paradox [11], reduced time to referral could also reflect more advanced disease; however, patients in this group had earlier/more locoregional disease. As such, there is a greater likelihood that direct access to diagnostic imaging and consultative services in the ED led to more timely referral. To decrease the primary care interval time in the community, primary care practitioners should have more direct and timely access to these services.

Lastly, we found that in both groups, most pre-diagnostic pathways were unique. Although it can be argued that pathways will vary depending on clinical presentation and medical history, this may also indicate a need for standardization. Given there are no referral guidelines in Québec for suspected lung cancer in primary care, an evidence-based guideline developed by Cancer Care Ontario could be adapted for local use [33].

To further inform improvement initiatives, qualitative inquiry to understand what contributes to the emergence of these pathways will be an important next step.

Strengths and Limitations

We present a single-center study based on a small sample of lung cancer patients from an urban setting. Given these constraints, we acknowledge generalizability concerns. Although provincial administrative healthcare databases would have allowed a broader sample, they lacked clinical data pertinent to our study (e.g., smoking history, presenting symptoms, disease stage) as well as granular data necessary to capture the complexity of cancer diagnostic pathways. As such, we used alternative data sources (clinic charts and patient self-reporting)—reasonable methods for any jurisdiction where administrative data may be incomplete or inaccessible. Additionally, we followed international standardized guidelines both in our study design and definitions (date of first presentation, date of referral, date of diagnosis, primary care interval) to ensure consistency with early cancer diagnosis literature [6].

Our sample demographics were similar to registry patient characteristics except for an overrepresentation of never smokers which may have been due to survival bias; patients diagnosed in 2015 had to survive two years to participate and better lung cancer survival has been reported among never smokers compared to ever-smokers [34]. There may have also been selection bias as the participation rate was 30% of the eligible sample despite efforts to contact patients in clinic and by phone. Similar recruitment challenges among lung cancer patients have been widely reported [35,36]. Despite this, model convergence was reached indicating good model–data fit and characteristics of the pre-diagnostic pathway groups coincided with the literature. Finally, recall periods varied depending on the length of the primary care interval and diagnosis date leading to potential recall bias. Several measures were used to mitigate this, including triangulation of data.

Notwithstanding these limitations, our study provides important evidence in an underresearched area of understanding cancer pathways in primary care.

## 5. Conclusions

Our study is the first in-depth look at the primary care interval of the lung cancer diagnostic pathway in Québec and contributes to a dearth of evidence in Canada on lung cancer diagnostic delays. We present several potential sources of delay and suggest associated initiatives to reduce avoidable delays in primary care. These include significant event audits, electronic consultation services, and referral guidelines.

## Figures and Tables

**Table 1:** Characteristics of patients included in the study.

Characteristic	Total Median (IQR) of Patients, * *n* = 50
Age at diagnosis, yr	66 (57.2–76.7)
Sex, female, *n* (%)	28 (56)
Smoking history, pack-yr	20.7 (0–42)
Number of comorbidities	1 (0–2)
Material deprivation index score ^†^	2 (1–4)
Social deprivation index score **^†^**	4 (2–5)
Primary care interval time, d	35 (9–100.7)
Referral source, *n* (%)	
Family physician	18 (36)
Emergency department	26 (52)
Non-respiratory specialist	6 (12)
Presenting symptoms, *n* (%)	
Cough	21 (42)
Shortness of breath	11 (22)
Hemoptysis	3 (6)
Chest pain	3 (6)
Back pain	4 (8)
Other **^‡^**	8 (16)
Stage of disease, *n* (%)	
Early	8 (16)
Locoregional	12 (24)
Advanced	30 (60)

Note: IQR = interquartile range. * Unless stated otherwise. ^†^ Classified in quintiles from least deprived (1) to most deprived (5). ^‡^ Includes weight loss, general weakness, paresthesia, abdominal pain, sinusitis, jugular vein thrombosis, and hoarseness.

**Table 2:** Statistical comparison of latent class models.

Latent Class Models	AIC
One-class	334.5149
Two-class	323.4714
Three-class	322.1228
Four-class	329.7299

**Table 3:** Characteristics of patients stratified by FP-centric and ED-centric groups.

Characteristic	FP-Centric; Median (IQR) of Patients, * *n* = 34	ED-Centric; Median (IQR) of Patients, * *n* = 16
Age at diagnosis, yr	65 (58.5–76.7)	67.5 (56.5–72)
Sex, female, *n* (%)	18 (52.9)	10 (62.5)
Smoking history, pack-yr	20 (0.4–35)	27.5 (0–49.2)
Number of comorbidities	1 (0.2–2)	0 (0–2)
Material deprivation index score ^†^	2 (1–3.7)	1 (1–3.5)
Social deprivation index score ^†^	4 (2–5)	4 (1.5–5)
Primary care interval time, d	45 (11.7–111.2)	22 (4.7–69.5)
Referral source, *n* (%)		
Family physician	17 (50)	1 (6.2)
Emergency department	13 (38.2)	13 (81.2)
Non-respiratory specialist	4 (11.6)	2 (12.4)
Presenting symptoms, *n* (%)		
Cough	16 (47.1)	5 (31.2)
Shortness of breath	8 (23.5)	3 (18.8)
Hemoptysis	2 (5.9)	1 (6.2)
Chest pain	2 (5.9)	1 (6.2)
Back pain	3 (8.8)	1 (6.2)
Other ^‡^	3 (8.8)	5 (31.2)
Stage of disease, *n* (%)		
Early	5 (14.7)	3 (18.8)
Locoregional	7 (20.6)	5 (31.2)
Advanced	22 (64.7)	8 (50)

Note: IQR = interquartile range. * Unless stated otherwise. ^†^ Classified in quintiles from least deprived (1) to most deprived (5). ^‡^ Includes weight loss, general weakness, paresthesia, abdominal pain, sinusitis, jugular vein thrombosis, and hoarseness.

## Supplementary Materials

The following are available online at https://www.mdpi.com/1718-7729/28/1/40/s1. Structured interview guide.
