# Supplementary material for: Lung Cancer Pre-Diagnostic Pathways from First Presentation to Specialist Referral
Source: Curr Oncol. 2021 Jan 11;28(1):378–89. doi: 10.3390/curroncol28010040 (PMC7903286; doi:10.3390/curroncol28010040)
Supplement: Supplementary file 1 [file curroncol-28-00040-s001.pdf]

Current Oncology

Supplemental Materials

# Lung Cancer Pre-Diagnostic Pathways from First Presentation to Specialist Referral

**Satya Rashi Khare , Sreenath Arekunnath Madathil , Gerald Batist , Peter Brojde Lung Cancer Group and Isabelle Vedel**

## Structured interview guide

***PART I***

*Note: Although this is a structured interview with specific data points, the interview itself will be conversational to facilitate recall of prior activities by 'talking-through' the pre-diagnostic pathway. The specific technique will be forward recall – start with first presentation in primary care and think forward to date of referral. A large calendar will be used to facilitate the interview and document the data.*

*The specific activities of interest include: 1) family physician visits (with whom the patient is registered), 2) visits to a walk-in clinic, 3) visits to an emergency department, 4) hospitalizations, 5) imaging tests (specifically CXR and CT), and 6) referrals to non-respiratory specialists.*

**INITIATION**:

1. Remind the participant(s) of the goals of the interview, projected length, and general topics of the interview.
    a. Suggested preamble: You were referred to a lung specialist on <REFERRAL DATE>. I am interested in what health care services you used – such as doctor appointments and tests – from when you had symptoms of lung cancer that made you see a doctor to the time you were referred to a lung specialist on <REPEAT DATE>. I have brought a calendar where we can record this information and hopefully make it a bit easier to remember the appointments you had.

2. Ask the participant(s) if he/she has their diary, appointment book, calendar, or anything else that could help remember appointments and activities.

3. Ask the participant if he/she has any questions before you start.

**INTERVIEW START**:

I would like to start with the first signs and symptoms of lung cancer that made you see a doctor. What were they and when did you see a doctor? <NOTE SIGNS AND SYMPTOMS ON THE CALENDAR ON THE DATE OF FIRST PRESENTATION, NOT MORE THAN 1 YEAR BEFORE THE REFERRAL DATE>

<IF THE PARTICIPANT HAS DIFFICULTY UNDERSTANDING THE QUESTION OR REMEBERING THEIR PRESENTING SIGNS AND SYMPTOMS, THEN USE THOSE LISTED IN THE CANCER CARE ONTARIO GUIDELINE AS A GUIDE>

Now, can you tell me what other appointments, tests, hospital visits, etcetera you had in this month (i.e. month of first presentation)? If an appointment or test was missed or cancelled, please tell me so I can put that on the calendar too. <NOTE ALL ACTIVITY ON THE CALENDAR ON THE DATES THEY OCCURRED – IF EXACT DATES ARE NOT KNOWN, USE AN APPROXIMATE DATE AND PLACE A QUESTION MARK BESIDE IT – USE THE SIDE COLUMNS FOR ADDED NOTES>

<CONTINUE THIS FOR EVERY MONTH UNTIL YOU REACH THE DATE OF REFERRAL>

**PROBES (if needed)**:

1. FAMILY PHYSICIAN VISITS
    a. Did you have a family doctor during this time? *IF YES*: How many times did you see your family doctor in this month?

2. WALK-IN CLINIC VISITS
    a. How many times did you go to a walk-in clinic in this month?

*<if the participant is unsure whether the clinic is considered a walk-in, ask if it is a clinic where they normally see their family physician if they have one>*

3. EMERGENCY ROOM VISITS
    a. How many times did you go to an emergency room in this month?

4. HOSPITALIZATIONS
    a. Were you ever hospitalized in this month? *IF YES*: How many times?

5. IMAGING TESTS
    a. Did you have any chest x-rays or CT scans in this month? *IF YES*: How many?

*< if the participant is unsure what these tests are, show pictures and briefly explain how the tests are done>*

6. REFERRALS TO NON-RESPIRATORY SPECIALISTS
    a. In this month, were you referred to see another specialist besides a lung specialist? *IF YES*: How many and what was their specialty (e.g. cardiology specialist, geriatric specialist, etcetera)? *<just to be certain it was a non-respiratory specialist>*

***PART II***

I have two final questions about your medical history.

1. What medications do you take for illnesses other than lung cancer and what do you take the medications for? *<ex. diabetes, hypertension, etc>*

2. Do you, or did you ever, smoke? *IF YES*: How many packs did you smoke per day and for how many years?

**CONCLUSION**:

1. Thank the participant for their contribution.
2. Explain how the project will proceed and how their information will be used.
3. Ask the participant if he/she has any questions before you conclude.

Version date: April 11, 2017
